# Non-Influenza and Non-SARS-CoV-2 Viruses Among Patients with Severe Acute Respiratory Infections in Tanzania: A Post-COVID-19 Pandemic Snapshot

**DOI:** 10.3390/v17081042

**Published:** 2025-07-25

**Authors:** Maria Ezekiely Kelly, Frank Msafiri, Francisco Averhoff, Jane Danda, Alan Landay, Azma Simba, Ambele Elia Mwafulango, Solomoni Mosha, Alex Magesa, Vida Mmbaga, Sandra S. Chaves

**Affiliations:** 1 National Public Health Laboratory, Dar es Salaam P.O. Box 9083, Tanzania; dandajane10@gmail.com (J.D.); ambeleeliah@yahoo.com (A.E.M.); 2 Management and Development for Health, Dar es Salaam P.O. Box 79810, Tanzania; frankmbulinyingi@yahoo.com; 3 Department of Microbiology and Immunology, Muhimbili University of Health and Allied Sciences, Dar es Salaam P.O. Box 65001, Tanzania; 4 Abbott Pandemic Defense Coalition, Abbott Park, IL 60064, USA; francisco.averhoff@abbott.com; 5 Department of Medicine, University of Texas Medical Branch Galveston, Galveston, TX 77555, USA; allanday@utmb.edu; 6 Department of Epidemiology, Ministry of Health, Dodoma P.O. Box 743, Tanzania; azmatan66@gmail.com (A.S.); moshitz@yahoo.com (S.M.); makundiv@yahoo.com (V.M.); 7 Department of Diagnostic, Ministry of Health, Dodoma P.O. Box 743, Tanzania; amagesa@yahoo.co.uk; 8 Foundation for Influenza Epidemiology, Fondation de France, 75008 Paris, France

**Keywords:** influenza, surveillance, post-COVID-19 pandemic, SARI, Tanzania

## Abstract

Respiratory pathogens are significant causes of morbidity and mortality worldwide. Since the emergence of SARS-CoV-2 in 2019 and the mitigation measures implemented to control the pandemic, other respiratory viruses’ transmission and circulation patterns were substantially disrupted. We leveraged the influenza hospitalization surveillance in Tanzania to understand the distribution of respiratory viruses shortly after nonpharmaceutical interventions (NPIs) were lifted. A total of 475 samples that tested negative for SARS-CoV-2 and influenza from March through May 2022 were included in this study. The samples were tested for 16 virus targets using Anyplex II RV16 multiplex assays. The findings indicate that most hospitalizations (74%) were among children under 15 years, with human bocavirus (HBoV) being the most prevalent (26.8%), followed by rhinovirus (RV, 12.3%), parainfluenza viruses (PIVs1–4, 10.2%), respiratory syncytial virus (RSV, 8.7%), adenovirus (AdV, 4.3%), and metapneumovirus (MPV, 2.9%). Notably, 54% of respiratory hospitalizations had no viruses detected. The findings highlight the broad circulation of respiratory viruses shortly after NPIs were lifted in Tanzania. Surveillance for respiratory pathogens beyond influenza and SARS-CoV-2 can inform public health officials of emerging threats in the country and should be considered an important pandemic preparedness measure at a global level.

## 1. Introduction

On 11 March 2020, the World Health Organization (WHO) officially designated COVID-19 as a pandemic [1,2]. Some countries reported that mitigation measures put in place to reduce the effects of the pandemic before vaccines were available contributed to the disruption in the circulation of many respiratory pathogens besides SARS-CoV-2 [3,4]. It was also noted that the implementation of NPIs during the pandemic to manage COVID-19 led to a reduction in children’s exposure to many pathogens, resulting in immunity debt [5]. While these measures successfully reduced the spread of SARS-CoV-2, they inadvertently lowered children’s exposure to a range of pathogens, which increased their vulnerability to infections when restrictions were lifted [6]. Nonetheless, in many African countries, the impact of the COVID-19 pandemic and NPIs on the circulation of other respiratory pathogens has not been well documented [7,8]. In 2016, Tanzania expanded its influenza sentinel surveillance system to capture other respiratory viruses in the country [9]. After the emergence of SARS-CoV-2, the surveillance program shifted priorities to focus solely on monitoring influenza and SARS-CoV-2. In 2022, as NPIs such as mask-wearing, school closures, and social distancing were lifted, the National Public Health Laboratory (NPHL) re-instituted testing of influenza and SARS-CoV-2 viruses in selected surveillance sentinel sites. We used this opportunity to explore whether other respiratory viruses were circulating in Tanzania after the lift of NPIs, using broad respiratory viral panel testing to assess, retrospectively, the presence of other viruses in those specimens that tested negative for influenza viruses or SARS-CoV-2, using a narrow time-window period.

## 2. Materials and Methods

Our study utilized stored samples (combined nasopharyngeal and oropharyngeal swabs) from five well-established influenza surveillance sites in Tanzania (Figure S1), collected from March to May 2022. These samples were collected following active, prospective screening for cases of all ages hospitalized with severe acute respiratory infection (SARI) [10]. Sites for the study were selected from diverse geographic locations, including the Kigoma region in the Western zone, Arusha and Manyara regions in the Northern zone, Dodoma region located in the central zone, and Dar es Salaam region in the coastal zone. Samples that tested negative for both influenza and SARS-CoV-2 were tested for other respiratory viruses. Following the manufacturer’s instructions, the Quick RNA Viral ™ Kit from Zymo Research, Irvine, CA, USA, was used for nucleic acid extraction. The LunaScript RT SuperMix Kit (New England Biolabs, Inc., Ipswich, MA, USA) was employed to generate cDNA from RNA extracted according to the manufacturer’s guidelines. A multiplex real-time reverse transcription polymerase chain reaction (RT-PCR) kit, Anyplex™ II RV16 detection (V1.1) from Seegene, Inc., Seoul, Republic of Korea, was utilized to detect the selected viruses, including adenovirus (AdV), parainfluenza virus 1 (PIV1), parainfluenza virus 2 (PIV2), parainfluenza virus 3 (PIV3), parainfluenza virus 4 (PIV4), rhinovirus (RV), human bocavirus 1/2/3/4 (HBoV), enterovirus (HEV), human metapneumovirus (HMPV), respiratory syncytial virus A (RSVA), and respiratory syncytial virus B (RSVB). The Anyplex™ II RV16 detection (V1.1) kit comes with an internal control (IC), which was added to each specimen during extraction to monitor the nucleic acid extraction and check for possible PCR inhibition. The IC is co-amplified with the target nucleic acid within specimens using the CFX 96 Real-Time PCR Thermal Cycler (Bio-Rad, Hercules, CA, USA). All procedures adhered to the manufacturer’s protocols. The amplification results were extracted from the machine and then exported for analysis using the Seegene Viewer software version 3.0.

Data analysis was conducted using STATA version 15 and GraphPad Prism version 10. In the descriptive analyses, categorical variables were presented using both numbers and percentages, while continuous variables were summarized using the median and interquartile range (IQR). Fisher’s exact test was used to determine the statistically significant association between categorical variables.

## 3. Results

A total of 852 samples were received from all five surveillance sites during the study period, of which 61 (7.1%) tested positive for influenza and 3 (0.3%) for SARS-CoV-2. Of the remaining 788 samples, only SARI cases were tested by the multiplex RT-PCR for our study, which was a total of 475 (60.3%). The median age of patients was one year (IQR: 0.33–23 years). Moreover, 44.8% of SARI cases (213/475) were aged 6 months to 14 years, representing the pediatric population in Tanzania. Males accounted for 53.5% (254/475) of all cases. Of the 475 samples tested, 219 (46.1%) were positive for at least one of the other tested viruses (Table 1).

**Figure 1:**
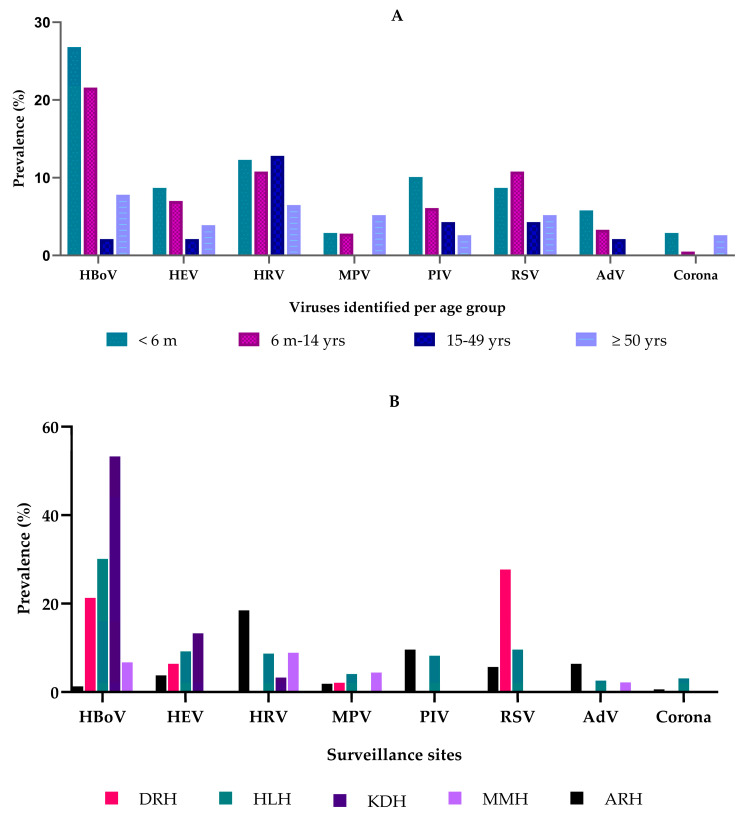
(**A**) Prevalence of non-influenza and non-SARS-CoV-2 viruses in SARI cases by age group, Tanzania. Abbreviations: adenovirus (AdV), parainfluenza virus (PIV1–4), rhinovirus (RV), human bocavirus 1/2/3/4 (HBoV), human enterovirus (HEV), metapneumovirus (MPV), and respiratory syncytial virus (RSVA&B). The color represents the age groups. (**B**) Prevalence of non-influenza and non-SARS-CoV-2 viruses in SARI cases across surveillance sites in Tanzania. Abbreviations: Arusha Regional Hospital (ARH), Dodoma Regional Hospital (DRH), Hydom Lutheran Hospital (HLH), Kibondo District Hospital (KDH), and Mwananyamala District Hospital (MMH). The color represents the surveillance sites.

Among those hospitalized, there were viruses detected in all ages, with distribution varying by age and region. Figure 1A shows HBoV as the most commonly detected virus among children < 6 months and children between 6 months and 14 years, followed by HRV and RSV in the same age group.

The site located in Western Tanzania, KDH, had the highest prevalence of HBoV cases (53.3%), followed by the central zone HLH site (30.1%) (Figure 1B). Rhinovirus cases were more common in the Northern zone ARH site (18.5%). Meanwhile, the central zone Dodoma site (DRH) had a significantly higher prevalence of RSV cases (27.7%).

Overall, 33.7% (160/475) had a single virus detected, while 12.4% (59/475) had more than one virus detected. The most common co-detection combinations were HBoV + HEV (5.9%; 13/219), HBoV + RSVA (3.7%; 8/219), HBoV + MPV (2.7%; 6/219), AdV + HRV (2.7%; 6/219), and RSVB + HEV (1.8%; 4/219). There were also a few instances of triple-virus combinations, with HBoV + RSVA + HEV (0.9%; 2/219) and MPV + HBoV + RSVB (0.5%; 1/219). Co-detection was more prevalent in children aged < 6 months (19.6%; 27/138), than in children aged 6 months to 14 years (12.7%; 27/213), adults aged 15–49 years (2.1%; 1/47) and adults > 50 years (5.2%; 4/47). There was only 23.7% of HBoV as a single detection (Table S1).

Table S2 indicates that there was no statistically significant difference between single infections and co-infections across age groups or facilities (*p* = 0.163, 0.223, respectively).

## 4. Discussion

In our short investigation, we found that among the hospitalized patients who tested negative for influenza viruses or SARS-CoV-2, almost half were positive for other viruses. Respiratory viruses were circulating throughout Tanzania and leading to healthcare utilization among people of all ages, particularly affecting the young, with the majority of cases among children (<15 years). The virus most frequently detected was HBoV, followed by HRV, RSV, PIV, and HEV. Co-detections with more than one respiratory virus were more commonly observed among young children aged ˃ 6 months (19.6%) and decreased substantially with age.

After the COVID-19 pandemic, the positivity rate for respiratory viruses other than influenza and SARS-CoV-2 still circulating was 46.1%. When compared to other studies, the same findings were observed, indicating a significant increase in the positivity rates from 37.1% and 44.05% during the pandemic to 68% after the pandemic [11,12]. Research conducted in China from 2019 to December 2023 also revealed a rise in respiratory pathogen positivity to 34.62% in 2023, up from 27.63% in 2021 and 24.38% in 2022 [13]. Other studies in the same area corroborate these findings, noting a 34.62% increase in the respiratory pathogen positivity rates following the easing of COVID-19 restrictions [14]. There was also a documented increase in the co-detection rates, as documented in one study from 5% to 16% [11]. This suggests that the implementation, followed by the lifting of NPIs, could have significantly affected the circulation patterns and interaction dynamics amongst respiratory viruses, but may have also affected the underlying immunity of the population no longer exposed to these viruses, especially at an early age [13,14,15,16].

This study also demonstrates a high prevalence of respiratory infections in children under 4 months affected by HBoV, HRV, PIV, and RSV. This supports the concept of immunity debt, as they were better protected when NPIs were implemented. Our findings are also consistent with other studies that indicate a high prevalence of pathogens like RSV among children just after NPIs were lifted [6,15,16].

Our study identified HBoV as the predominant virus, with notable detection rates in children under 14 years. HBoV has appeared as an emerging pathogen in the 21st century that can cause respiratory diseases in children, including the common cold, acute otitis media, pneumonia, bronchiolitis, and asthma exacerbations [17,18]. However, its DNA can persist in airway secretions for months after an acute infection, making it difficult to diagnose an acute episode of the HBoV infection based on standard PCR tests alone [19,20,21]. Interestingly, there has also been a reported surge in HBoV cases in children during the COVID-19 pandemic, underscoring the importance of understanding and monitoring such respiratory viral infections in the context of healthcare surge capacity [22,23]. Nonetheless, while HBoV has often been detected in respiratory samples, its role as a primary pathogen versus an incidental finding continues to be studied.

Our investigation identified co-detection with multiple viruses in 26.9% of all the cases enrolled. Notably, the co-occurrence of HBoV with other respiratory viruses, such as HEV, RSV, and HMPV, was particularly prominent. This finding aligns with previous research, as multiple studies have documented similar co-infection rates [17,24]. The rates of the simultaneous detection of HBoV with other respiratory viruses have been reported to range from 37% to 76% in various studies [17,25,26]. We found a single HBoV detection in 23.7% of our hospitalized cases.

Our data show that respiratory viruses other than influenza and SARS-CoV-2 play an important role in the burden caused by respiratory illnesses in Tanzania. Still, more than half of the respiratory hospitalizations showed no identified viral pathogen. However, our list of viral targets was limited by the panel used in this study, and the investigation of bacterial pathogens was not included. Understanding the circulation of respiratory viruses and how they affect different population groups can help prioritize resources or identify unmet medical needs that should be addressed by research and development. Tanzania, like many other African nations, has limited resources and competing public health priorities, but considerations regarding year-round integrated respiratory surveillance and the broadening of laboratory testing to other respiratory viruses beyond influenza and SARS-CoV-2 should be considered, as it can support stakeholders and policymakers in their decision-making for limited healthcare resources. As an example, RSV is currently a vaccine-preventable disease, and most of the associated mortality among children occurs in low- and middle-income countries, where vaccine access is limited [27]. Surveillance could assist public health prioritizations, leveraging new therapeutics and vaccines to reduce morbidity and mortality equitably. Investments in diagnostics and surveillance are needed to inform these decisions.

Our study had several limitations that need to be considered. First, due to a limited respiratory pathogen panel, we potentially overlooked other causes of SARI hospitalizations, including bacterial pathogens. We also did not have data on clinical outcomes from the surveillance cases and could not discuss disease severity. In addition, the study was confined to five sites over three months, limiting the generalizability of our findings. A more extended study with a wider geographic coverage could have enhanced our understanding of the circulating viruses in the post-pandemic period in Tanzania. Moreover, we do not know if the NPI measures put in place in the country were successful in curbing the impact of SARS-CoV-2, and how much that impacted the circulation of the viruses. Despite these constraints, our study provides valuable insights into the non-influenza and non-SARS-CoV-2 viruses as important contributors to SARI in hospitalized patients, particularly in children, within Tanzania.

## 5. Conclusions

Leveraging established surveillance systems, like influenza sentinel surveillance, can be a cost-efficient strategy for monitoring the circulation of a broad range of respiratory viruses, assessing potential interactions among circulating viruses, and helping to identify emerging public health threats in the future.

## Figures and Tables

**Table 1:** Demographic characteristics of patients with severe acute respiratory illness overall and among those with ≥1 virus detected, N = 475, Tanzania, March to May 2022.

Variables	Total Cases (N = 475)		Negative Cases (256; 53.9%)		Cases with ≥1 Virus Detected (N = 219; 46%)	
	***n***	**%**	***n***	**%**	***n***	**%**
Age group						
<6 months	138	29	60	23.4	78	35.6
6 months–14 years	213	44.8	106	41.4	107	48.9
15 years–49 years	47	9.9	35	13.7	12	5.5
≥50 years	77	16.2	55	21.5	22	10
Sex						
Male	254	53.5	144	56.2	110	50.2
Female	221	46.5	112	43.7	109	49.8
Presence of comorbidity						
Any comorbidities	51	10.7	37	14.4	14	6.4
Diabetes	3	0.6	2	0.8	1	0.5
Heart disease (excluding hypertension)	27	5.7	22	8.6	5	2.3
Hypertension	27	5.7	21	8.2	6	2.7
HIV	10	2.1	7	2.7	3	1.4
Asthma	3	0.6	1	0.4	2	0.9
Facility						
Arusha Regional Hospital (ARH)	157	33	97	37.9	60	27.4
Dodoma Regional Hospital (DRH)	47	9.9	24	9.4	23	10.5
Hydom Lutheran Hospital (HLH)	196	41.3	87	34.0	109	49.8
Kibondo District Hospital (KDH)	30	6.3	11	4.3	19	8.7
Mwananyamala District Hospital (MMH)	45	9.5	37	14.4	8	3.7
Month of Hospitalization						
March	180	37.9	105	41.0	75	34.2
April	175	36.8	79	30.9	96	43.8
May	120	25.3	72	28.1	48	21.9

## Data Availability

The data is unavailable due to ethical restrictions.

## Supplementary Materials

The following supporting information can be downloaded at: https://www.mdpi.com/article/10.3390/v17081042/s1, Figure S1: Selected surveillance sites for the SARI study. The map illustrates 26 administrative regions in mainland Tanzania. The large round red dot indicates the selected sites for the study. Map created with QGIS 3.24.1. All shapefiles are openly available sources (https://www.nbs.go.tz/statistics/topic/gis (accessed on 25 May 2025)). The shapefiles were based on the 2012 population and housing census, but for this study, the shapefile has been modified to capture all regional and district information; Table S1: Viruses isolated from SARI cases from March to May 2022, based on age group; Table S2: Positive cases from March to May 2022, categorized by age group and facility.
