# Supplementary material for: Non-Influenza and Non-SARS-CoV-2 Viruses Among Patients with Severe Acute Respiratory Infections in Tanzania: A Post-COVID-19 Pandemic Snapshot
Source: Viruses. 2025 Jul 25;17(8):1042. doi: 10.3390/v17081042 (PMC12390727; doi:10.3390/v17081042)
Supplement: Supplementary file 1 [file viruses-17-01042-s001.zip › Table S1 Viruses isolated from SARI cases from March to May 2022 based on age group.pdf]

**Table S1. Viruses isolated from SARI cases from March to May 2022, based on age group**

| Isolated viruses | (< 6 m)<br>N= 138<br>n (%) | (6 m – 14 y)<br>N = 213<br>n (%) | (15 – 49 y)<br>N=47<br>n (%) | (≥ 50 y)<br>N=77<br>n (%) |
|---|---|---|---|---|
| Negative | 60 (43.5) | 106 (49.8) | 35 (74.5) | 55 (71.4) |
| Single Infection | 51 (37) | 80 (37.5) | 11 (23.4) | 18 (23.4) |
| Co-infection | 27 (19.5) | 27 (12.7) | 1 (2.1) | 4 (5.2) |
| AdV | 4 (2.9) | 2 (0.9) | 1 (2.1) | 0 |
| AdV, HRV | 3 (2.2) | 3 (1.4) | 0 | 0 |
| AdV, PIV3 | 1 (0.7) | 0 | 0 | 0 |
| AdV, PIV2, HRV | 0 | 1(0.5) | 0 | 0 |
| HBoV | 18 (13.0) | 30 (14.1) | 1 (2.1) | 3 (3.9) |
| HBoV, 229E | 2 (1.4) | 1 (0.5) | 0 | 0 |
| HBoV, HEV | 6 (4.3) | 6 (2.8) | 0 | 1 (1.3) |
| HBoV, OC43 | 2 (1.4) | 0 | 0 | 0 |
| HBoV, RSVA | 2 (1.4) | 6 (2.8) | 0 | 0 |
| HBoV, RSVA, HEV | 2 (1.4) | 0 | 0 | 0 |
| HBoV, RSVB | 1 (0.7) | 2 (0.9) | 0 | 1() |
| HEV | 3 (2.2) | 7 (3.3) | 0 | 1 (1.3) |
| HRV | 11 (7.9) | 17 (8.0) | 6 (12.8) | 5 (6.5) |
| MPV | 0 | 3 (1.4) | 0 | 2 (2.6) |
| MPV, HBoV | 3 (2.2) | 1 (0.5) | 0 | 2 (2.6) |
| MPV, HboV, RSVB | 1 (0.7) | 0 | 0 | 0 |
| MPV, HEV | 0 | 1 (0.5) | 0 | 0 |
| MPV, RSVB | 0 | 1 (0.5) | 0 | 0 |
| NL63 | 0 | 0 | 0 | 1 (1.3) |
| OC43 | 0 | 0 | 0 | 1 (1.3) |
| PIV1 | 0 | 1 (0.5) | 1 (2.1) | 0 |
| PIV1, HRV | 1 (0.7) | 0 | 0 | 0 |
| PIV2 | 3 (2.2) | 1 (0.5) | 0 | 1 (1.3) |
| PIV2, HRV | 0 | 1 | 0 | 0 |
| PIV3 | 1 (0.7) | 0 | 1 (2.1) | 0 |
| PIV3, HRV | 1 (0.7) | 2 (0.9) | 0 | 0 |
| PIV4 | 6 (4.3) | 6 (2.8) | 0 | 1 (1.3) |
| PIV4, AdV | 0 | 2 (0.9) | 0 | 0 |
| PIV4, HRV | 1 (0.7) | 0 | 0 | 0 |
| RSVA | 1 (0.7) | 4 (1.9) | 1 (2.1) | 0 |
| RSVB | 4 (2.9) | 9 (4.2) | 0 | 3 (3.9) |
| RSVB, HEV | 1 (0.7) | 1 (0.5) | 1 (2.1) | 1 (1.3) |

**Table S2. Positive cases from March to May 2022, categorized by age group and facility**

| Variables | Total Positive cases 219 | | Single infection (160;73.0%) | | Co-infection (59;26.9%) | | P value* |
|---|---|---|---|---|---|---|---|
| | n | % | n | % | n | % | |
| **Age group** | | | | | | | |
| < 6 months | 78 | 35.6 | 51 | 31.9 | 27 | 45.8 | |
| 6 months – 14 years | 107 | 48.9 | 80 | 50.0 | 27 | 45.8 | 0.163 |
| 15 years – 49 years | 12 | 5.5 | 11 | 6.9 | 1 | 1.7 | |
| ≥ 50 years | 22 | 10.0 | 18 | 11.2 | 4 | 6.8 | |
| **Facility** | | | | | | | |
| Arusha Regional Hospital (ARH) | 60 | 27.4 | 45 | 28.1 | 15 | 25.4 | |
| Dodoma Regional Hospital (DRH) | 23 | 10.5 | 19 | 11.9 | 4 | 6.8 | |
| Hydom Lutheran Hospital (HLH) | 109 | 49.7 | 73 | 45.6 | 36 | 61.0 | 0.223 |
| Kibondo District Hospital (KDH) | 19 | 8.7 | 17 | 10.6 | 2 | 3.4 | |
| Mwananyamala District Hospital (MMH) | 8 | 3.7 | 6 | 3.8 | 2 | 3.4 | |

*P-value represents a statistical test comparing single infection to co-infection detected using the Fisher's exact test
