# Supplementary material for: Non-Influenza and Non-SARS-CoV-2 Viruses Among Patients with Severe Acute Respiratory Infections in Tanzania: A Post-COVID-19 Pandemic Snapshot
Source: Viruses. 2025 Jul 25;17(8):1042. doi: 10.3390/v17081042 (PMC12390727; doi:10.3390/v17081042)
Supplement: Supplementary file 1 [file Figure S1 Selected sites for SARI study.pdf › Figure S1 Selected sites for SARI study.pdf]

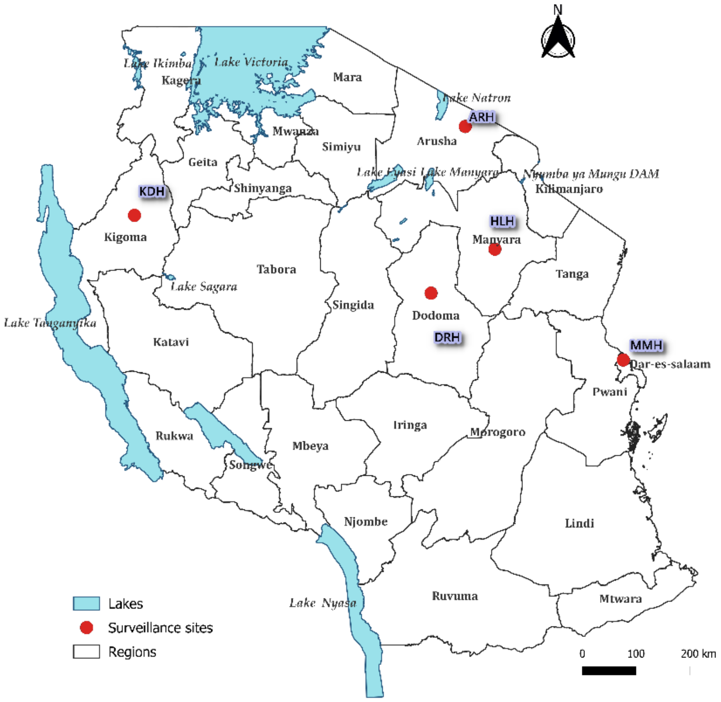

Figure S1: Selected surveillance sites for the SARI study

The map illustrates 26 administrative regions in mainland Tanzania. The large round red dot indicates the selected sites for the study. Map created with QGIS 3.24.1. All shapefiles are openly available sources (https://www.nbs.go.tz/index.php/en/census-surveys/gis/385-2012-phc-shapefiles-level-one-and-two).

The shapefiles were based on the 2012 population and housing census, but for this study, the shapefile has been modified to capture all regional and district information.
